# Supplementary material for: Whole-Genome Sequencing and Genetic Diversity of Human Respiratory Syncytial Virus in Patients with Influenza-like Illness in Sicily (Italy) from 2017 to 2023
Source: Viruses. 2024 May 26;16(6):851. doi: 10.3390/v16060851 (PMC11209242; doi:10.3390/v16060851)

**Figure S1.** NGS sequencing workflow for hRSV-A

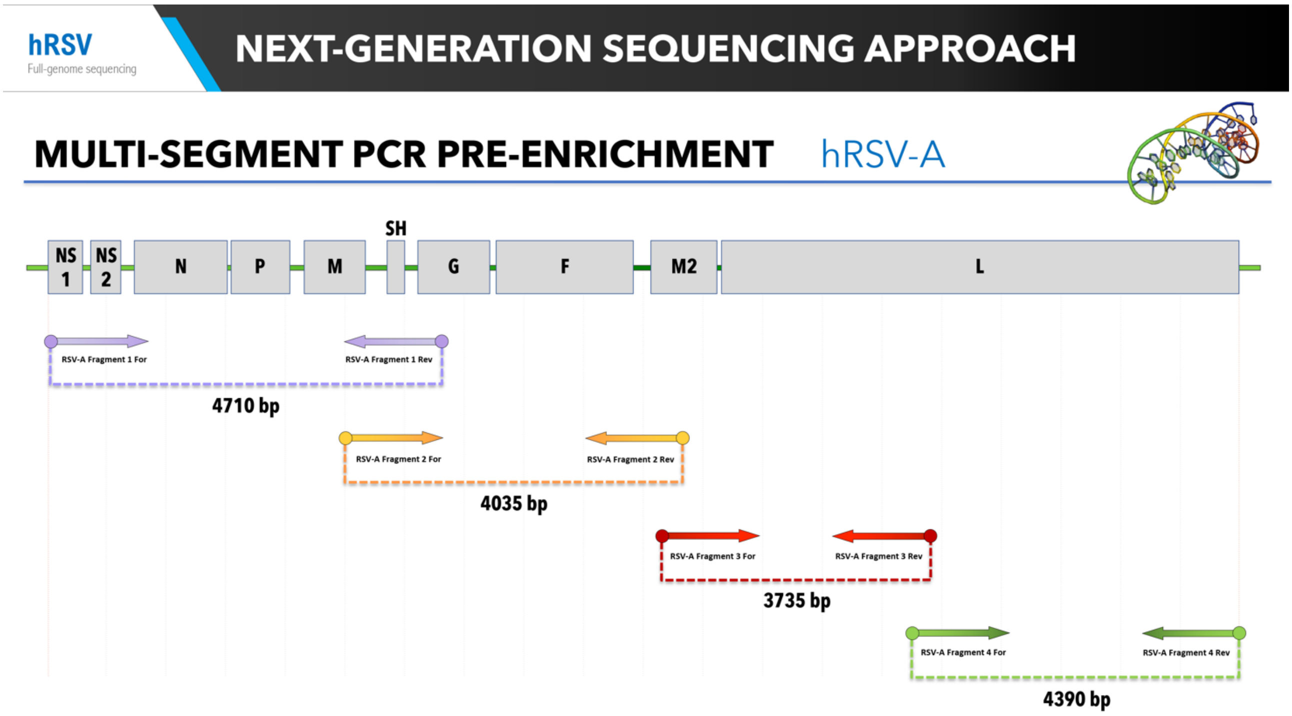


**Figure S2.** NGS sequencing workflow for hRSV-B

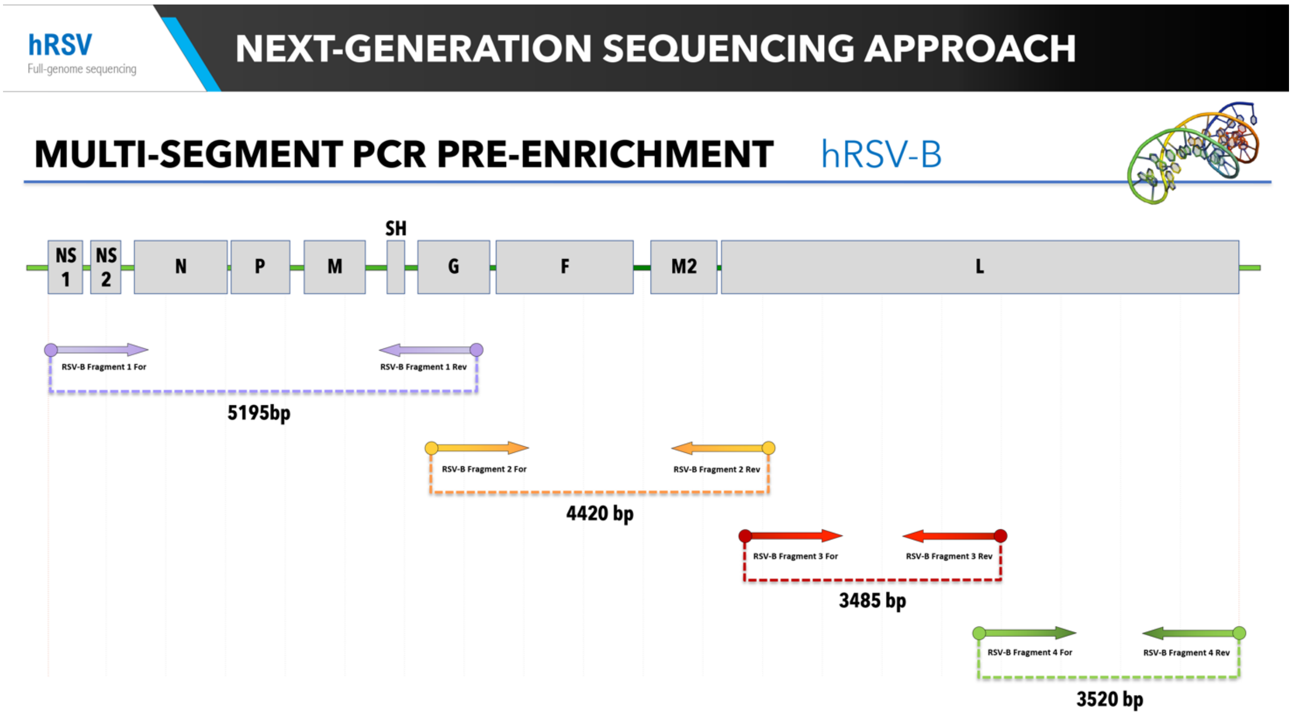

**Table S1.** Primers used for whole-genome sequencing of hRSV.

**hRSV-A**

| Primer | Sequence (5′ to 3′) |
|---|---|
| hRSV-A Fragment 1 For | AAAAATGCGTACWACAAACTTGC |
| hRSV-A Fragment 1 Rev | GTTGGTCCTTGGTTTTGGAC |
| hRSV-A Fragment 2 For | CACAGTGACTGACAACAAAGGAG |
| hRSV-A Fragment 2 Rev | GCTCATRGCAACACATGC |
| hRSV-A Fragment 3 For | CGAGGTCATTGCTTGAATGG |
| hRSV-A Fragment 3 Rev | CACCACCACCAAATAACATGG |
| hRSV-A Fragment 4 For | AGGGTGGTGTCAAAAACTATGG |
| hRSV-A Fragment 4 Rev | ACGAGAAAAAAAGTGTCAAAAACT |

**hRSV-B**

| Primer | Sequence (5′ to 3′) |
|---|---|
| hRSV-B Fragment 1 For | AAAAATGCGTACTACAAACTTGC |
| hRSV-B Fragment 1 Rev | CRAAGTTGAACACTTCAAAATGG |
| hRSV-B Fragment 2 For | AAGGGTTAGCCCATCCAAMC |
| hRSV-B Fragment 2 Rev | TGCTRAGGCTGATGTCTTTCC |
| hRSV-B Fragment 3 For | GTCCTCGTCTGARCAAATTGC |
| hRSV-B Fragment 3 Rev | TAGGTCCTCTTTCACCACGAG |
| hRSV-B Fragment 4 For | GAGGGATCCACAGGCTTTAGG |
| hRSV-B Fragment 4 Rev | ACGAGAAAAAAGTGTCAAAAACT |

**Table S2.** Seasonal distribution of hRSV whole-genome sequences, according to hRSV subgroup. Period: 2017–2023 (% by row).

| | **Total** | **hRSV-A** | **hRSV-B** |
|---|---|---|---|
| **hRSV whole-genome sequences [n (%)]** | 153 | 73 (47.7) | 80 (52.3) |
| **Surveillance season** | | | |
| ***2017-2018*** | 22 | 11 | 11 |
| ***2018-2019*** | 28 | 9 | 19 |
| ***2019-2020*** | 32 | 23 | 9 |
| ***2020-2021*** | 1 | 0 | 1 |
| ***2021-2022*** | 31 | 12 | 19 |
| ***2022-2023*** | 39 | 18 | 21 |

**Figure S3a**. Amino acid variation frequencies detected in the NS1 protein open reading frames of hRSV-A and hRSV-B Sicilian strains. Proportion of mutated AA positions: 5.8% for hRSV-A, 5.8% for hRSV-B.

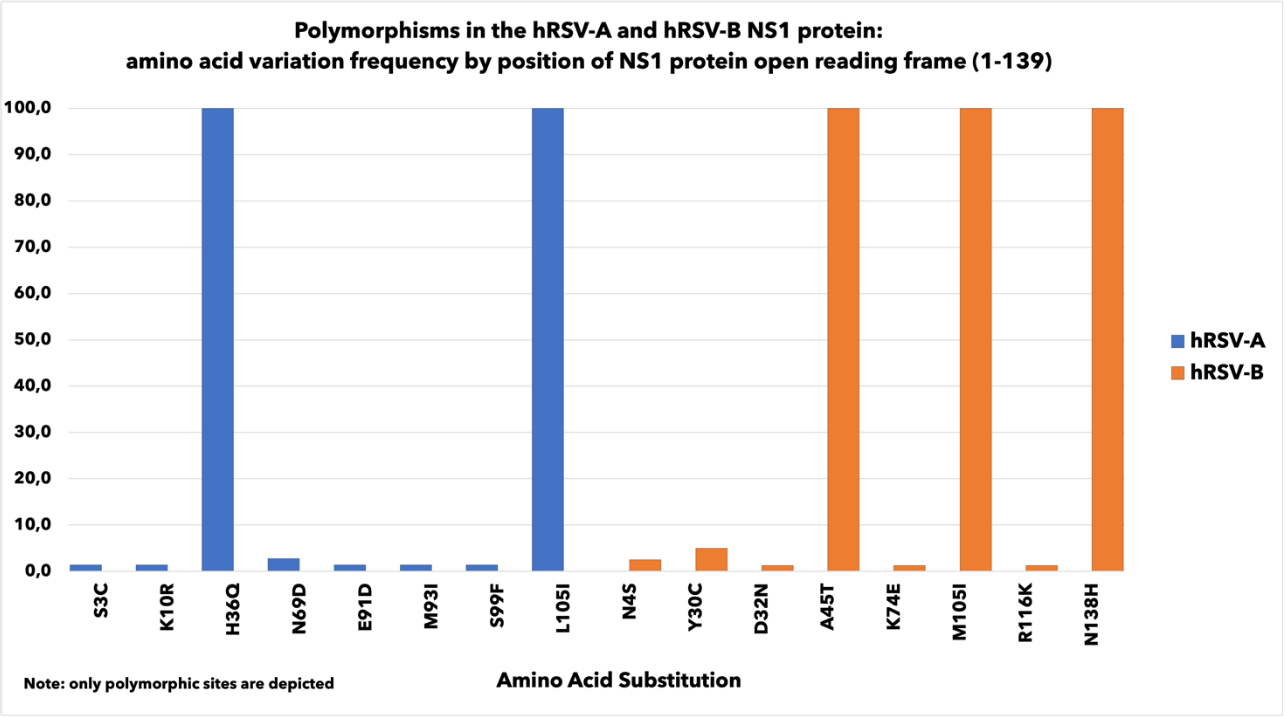


**Figure S3b**. Amino acid variation frequencies detected in the NS2 protein open reading frames of hRSV-A and hRSV-B Sicilian strains. Proportion of mutated AA positions: 6.5% for hRSV-A, 11.3% for hRSV-B.

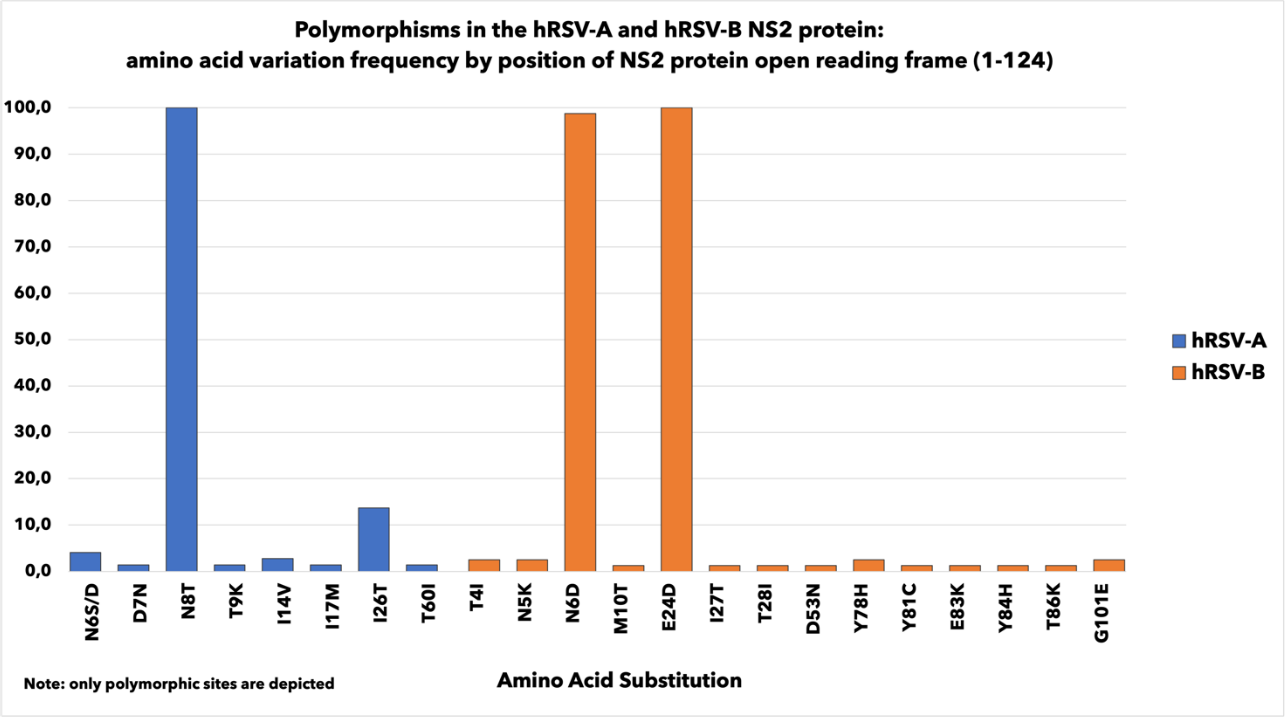

**Figure S4a**. Amino acid variation frequencies detected in the N protein open reading frames of hRSV-A and hRSV-B Sicilian strains. Proportion of mutated AA positions: 1.5% for hRSV-A, 0.8% for hRSV-B.

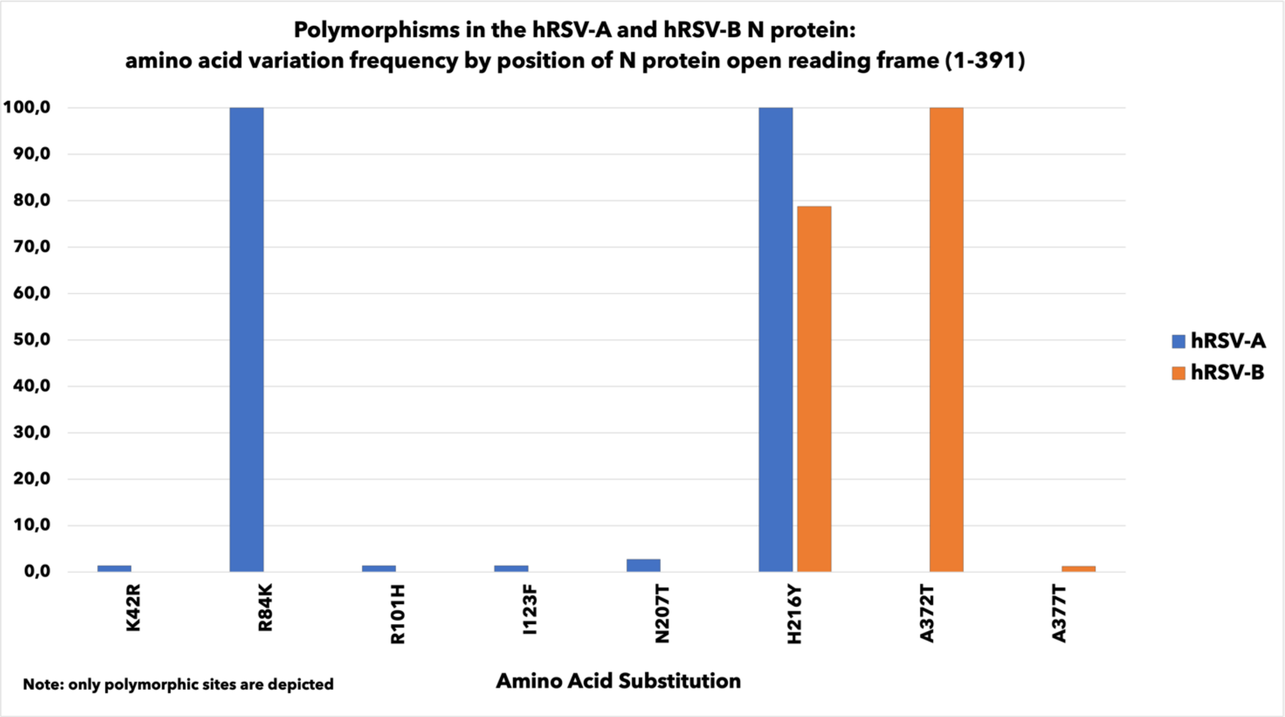


**Figure S4b**. Amino acid variation frequencies detected in the P protein open reading frames of hRSV-A and hRSV-B Sicilian strains. Proportion of mutated AA positions: 4.6% for hRSV-A, 5.0% for hRSV-B.

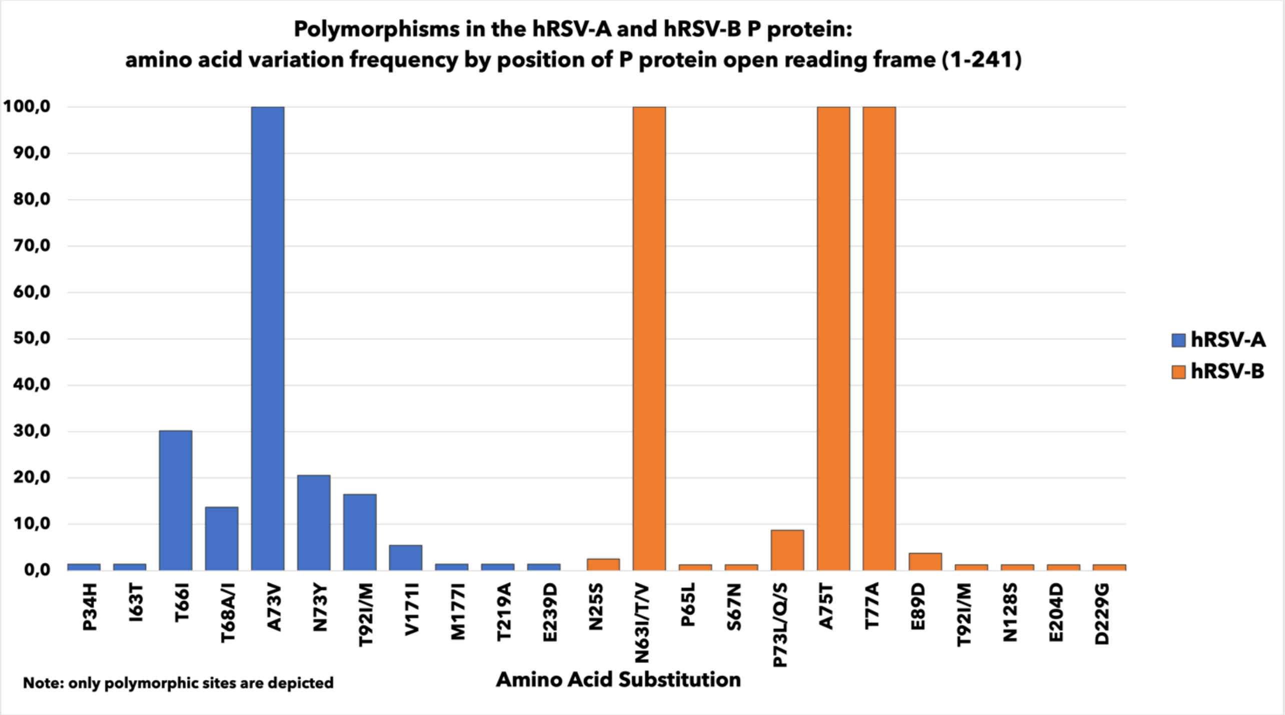

**Figure S5a**. Amino acid variation frequencies detected in the M protein open reading frames of hRSV-A and hRSV-B Sicilian strains. Proportion of mutated AA positions: 3.5% for hRSV-A, 2.0% for hRSV-B.

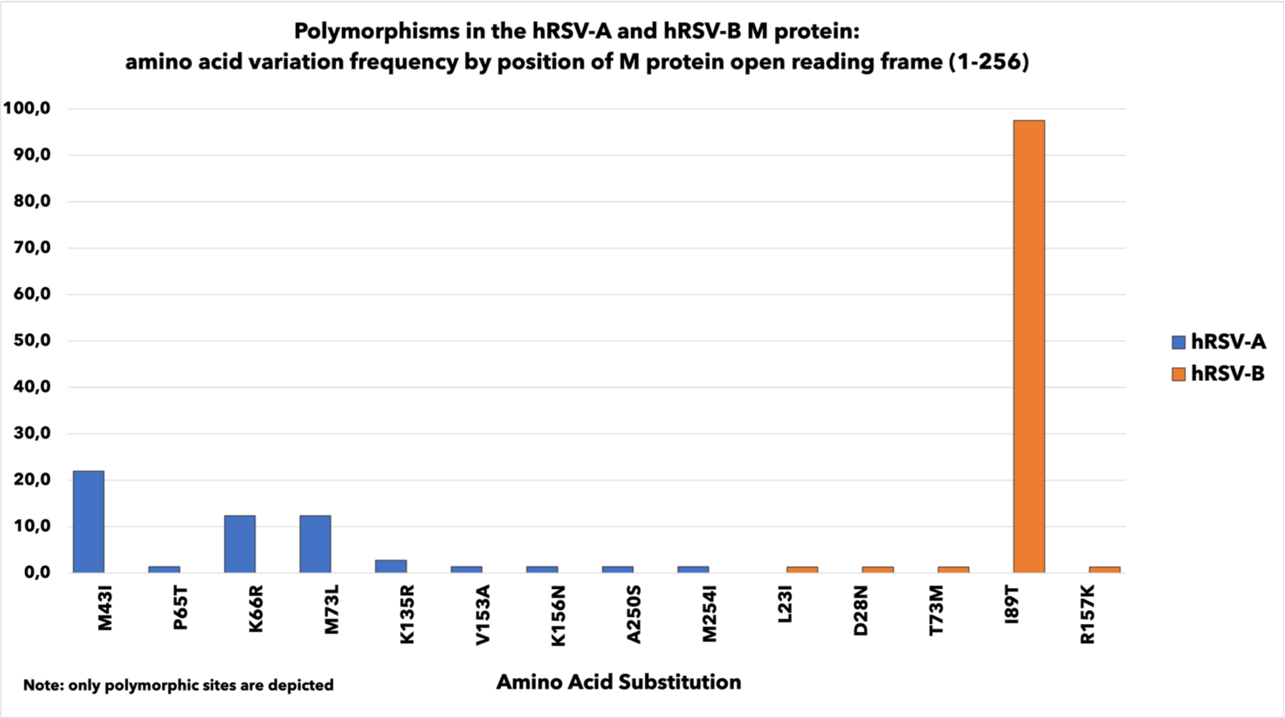


**Figure S5b**. Amino acid variation frequencies detected in the SH protein open reading frames of hRSV-A and hRSV-B Sicilian strains. Proportion of mutated AA positions: 10.8% for hRSV-A, 16.9% for hRSV-B.

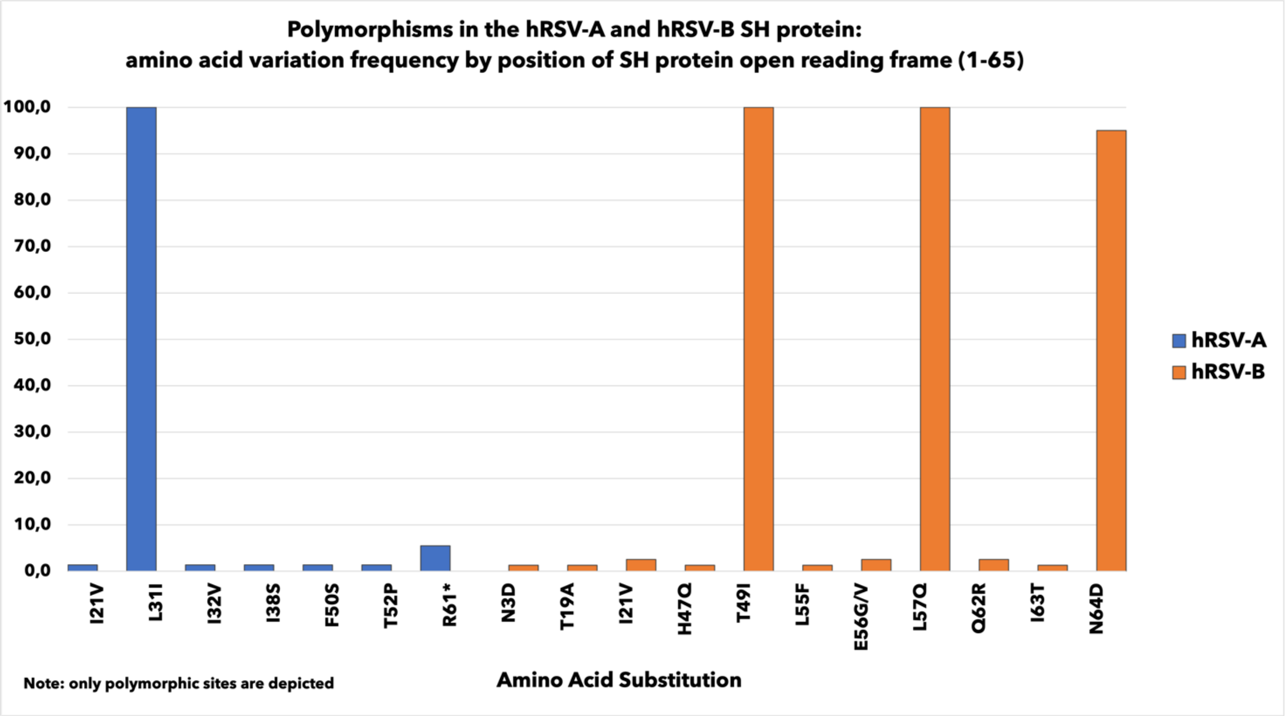

**Figure S6a**. Amino acid variation frequencies detected in the M2-1 protein open reading frames of hRSV-A and hRSV-B Sicilian strains. Proportion of mutated AA positions: 6.1% for hRSV-A, 5.1% for hRSV-B.

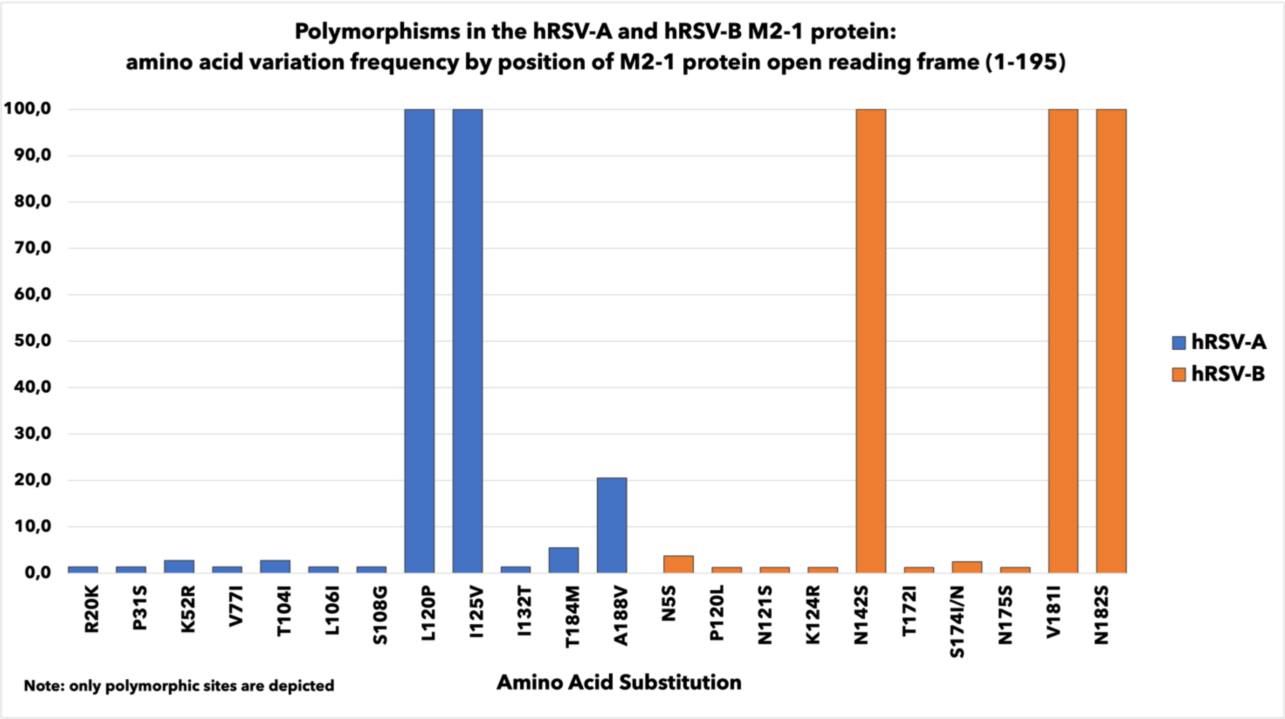


**Figure S6b**. Amino acid variation frequencies detected in the M2-2 protein open reading frames of hRSV-A and hRSV-B Sicilian strains. Proportion of mutated AA positions: 22.6% for hRSV-A, 15.0% for hRSV-B.

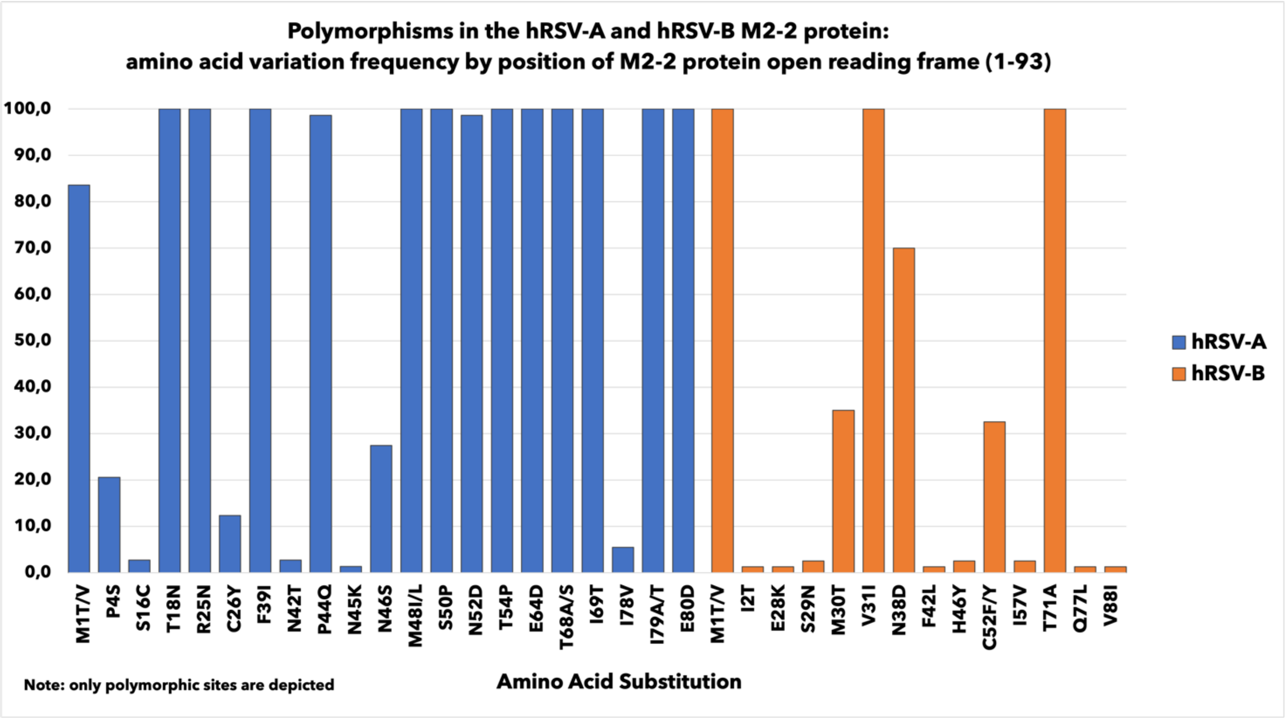

**Figure S7**. Amino acid variation frequencies detected in the L protein open reading frame of hRSV-A and hRSV-B Sicilian strains. Proportion of mutated AA positions: 5.3% for hRSV-A, 5.3% for hRSV-B.

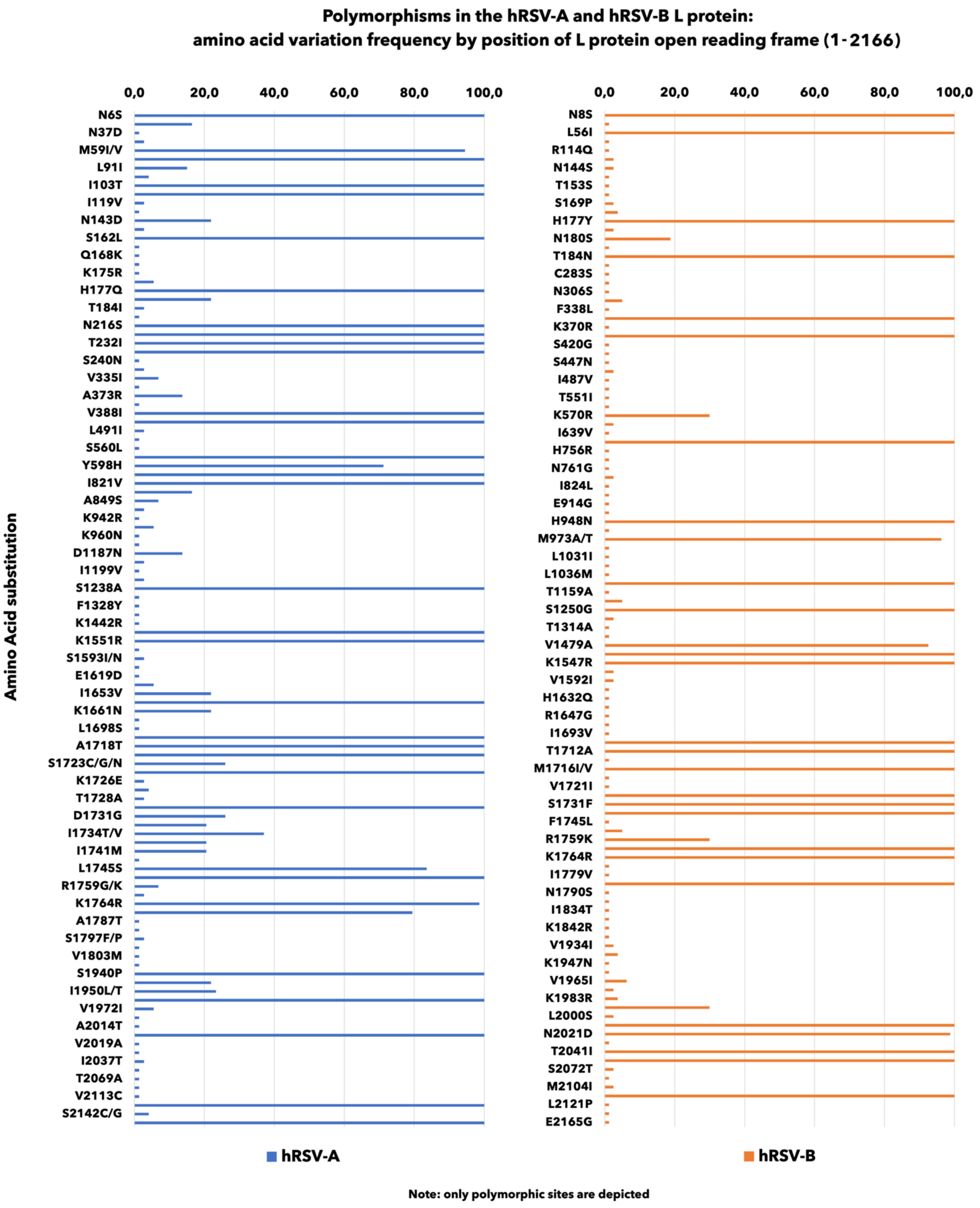

Supplement: Supplementary file 1 [file viruses-16-00851-s001.zip › Supplementary materials.pdf]
